# Abstract of the Proceedings of the Buffalo Medical Association

**Published:** 1861-11

**Authors:** J. F. Miner

**Affiliations:** Sec’y.


ART. II.—*Abstract of the Proceedings of the Buffalo Medical Association.*

TUESDAY EVENING, Oct. 1, 1861.

The Association met at the usual hour.

Present—Dr. C. C. F. Gay, President, in the Chair; Drs. Samo, Eastman, Rochester, Ring, Wyckoff, White, Strong, Congar, Miner and Gould.

Minutes of the last meeting read and approved.

*Voted*, that Dr. T. M. Johnson, of Buffalo, be invited to participate in the proceedings of the Association until he shall be able to complete his membership with the Erie County Society.

*Voted*, that P. Tertius Kempson, M. D., of Fort Erie, Canada West, be invited to participate in the proceedings of the Association.

A salivary calculus was shown the Society by *Prof. White*, who remarked that, it had been kindly sent him by Dr. G. A. Irvine, of Warren, Pa. The specimen was about one-half inch in length, cylindrical in form, and about four lines in diameter. It was only a part of the entire formation, the remaining third or thereabouts, was not removed until some time after this was discharged, and was lost by the wife of the patient. The concretion is composed chiefly of carbonate of lime, held together by animal tissue. It is not a little remarkable that so large a stone could form in the small ducts of the sublingual, and that too without inconvenience to the patient. How long it may have remained in its bed without producing irritation, does not appear. At length inflammatory action was excited, an abscess formed, which ruptured after a day or two, exposing the calculus to view. Dr. Irvine with a pair of forceps, easily removed the large cylinder here shown, and after a day or two the remaining portions. The patient has entirely recovered, leaving but slight trace of its consequences in the floor of the mouth.

*Prof. White* then called the attention of the Society to the use of the solution of the per sulphate of iron in "Bright's disease." A patient who had been subjected to all the ordinary remedies for this intr actible disease by Dr. Ellenwood, of Pembroke, was last April sent to Dr. White for examination and suggestions. The patient thirty-six years old, blacksmith by trade, originally of vigorous constitution, but now presented all the symptoms of persistent albuminuria. The feet and legs were enormously swollen, and the œdema extended above the hips with largely distended abdomen, the arms and hands would pit ; respiration labored ; complains of dull pain in the lumbar region on both sides ; micturition frequent, is obliged to rise several times in the course of the night for that purpose ; specific gravity of urine 1014 coagulable by heat and nitric acid. The urine upon standing deposits a sediment which, when placed under the microscope by my colleague, Prof. Rochester, who examined the patient with me, was found to contain amorphus urate of ammonia, with numerous tube-casts. The patient suffered greatly with palpitation, but the cardiac sounds were normal ; is greatly exhausted and easily overcome by the slightest exertion.

The treatment during the first month after he came under observation, consisted in an effort to improve his hygienic condition by tonics, air and the free use of digitalis and bi-tartrate of potash, glycerine and tannin. Not finding him improving under the use of the ordinary remedies, it was determined to make use of the solution of the per sulphate of iron—commenced with fifteen drops three times daily, and continued passive exercise in the open air with nutritious diet. Upon his return for re-examination after one month there was a manifest improvement in his general appearance and spirits. The dose was now increased to thirty drops four times daily, and the measures for invigoration of the general system continued.

*September 15th.*—The iron has been taken now for almost three months and he returns to town to-day, for a supply of the medicine, so much better that he wishes to increase the amount taken to forty drops four times daily. He works a part of the time at his trade, and bears exercise without exhaustion. The œdema in the lower extremities, is greatly lessened and the size of the abdomen greatly reduced. Upon testing his urine with heat and nitric acid, no albumen could be detected. He promised me that he would leave some of his urine with Prof. Rochester for microscopic examination, which promise he omitted to fulfil, in his haste to return by "next train." I regret also, that I was so much occupied at the time of his visit as not to be able to ascertain its specific gravity. You perceive therefore, gentlemen, that the report of the case ise xceedingly imperfect, indeed, may be said to be now in progress. Dr. White said his chief object in referring to the case, was to call the attention of the members to this form of iron as a remedy for this disease. He was aware that one "swallow did not make a summer," and did not claim that the very great improvement in Hamilton's condition should be set down to the credit of the iron. But in the absence of a better remedy he hoped the members would give it a fair trial if opportunity presented, and promised to inform the Society from time to time, of the condition, and final result, of the case now imperfectly narrated.

*Prof. Rochester* said that he had several times reported cases, treated both in hospital and private practice, in which tannin was very useful in this form of disease. Has regarded tannin as superior to gallic acid, which Prof. Hadley explains, by saying that the tannic acid is probably converted into gallic acid in the stomach, and the recently prepared article acts better while fresh. Would mention an instance corroborating Prof. White's opinion of the efficacy of the per sulphate of iron in Bright's disease. He was consulted in May last, by a patient for whom he had frequently prescribed, during seven or eight years, for malarious diseases ; he was fifty-five years old, of regular habits, and by occupation a night watchman. On this occasion, learned that he had been suffering for six months or more with œdema of the lower extremities, that his face and hands were often puffy ; that he was frequently giddy, and that his general health was much impaired. A

portion of urine was examined; microscopic examination of the sediment, disclosed fatty granules, and fatty and fibrinous casts of the tubuli uriniferi. Heat and nitric acid converted the contents of a test tube into almost solid albumen. The patient was placed upon the bi-tartrate of potash in purgative doses, and upon three grains of tannin every six hours dissolved in one drachm of glycerine. Rapid amendment took place, but after the persistent employment of these agents for two months, a considerable amount of albumen was always found in the urine. Ten drops of Squibb's solution of the per sulphate of iron, was now directed three times daily, in addition to the other remedies ; care being taken that the tannin and iron should not mingle in the stomach. After continuing the iron for one month, no albumen was discoverable, nor could any casts of the uriniferous tubes be detected. The patient still takes the iron twice daily, but is apparently quite restored.

*Dr. Miner* said he had a case under treatment which interested him, and had in some respects a bearing upon the topics presented by Drs. White and Rochester.

Visited Mrs. ——, September 23d, upon examination found general anasarca ; the labia majora immensely distended, which symptom had appeared suddenly, and was the cause of being called at this time ; respiration labored ; urine scanty ; bowels constipated ; vomiting had been constant for some time past, every thing immediately rejected from the stomach, until for the last two days it had greatly abated, improvement in this respect was attributed to the use of kreosote ; menstruation suppressed for the last eight months, and she supposed herself pregnant ; no fœtal heart sounds audible, no motion, and the uterine tumor indistinctly felt, on account of effusion into abdominal cavity ; urine highly albuminous, so much as to hardly subside sufficiently in the test tube to leave any portion clear. Prescribed tannin, three grains every four hours, and saline cathartics.

*September* 29.—The œdema of the labia had so greatly subsided that a speculum was easily introduced, and the membranes were ruptured, a bloody serum escaping in small quantity ; labor commenced in six hours and soon terminated, the product being a fœtus of about 8 months, which had probably been dead for a few days.

This patient complained of great pain in the head, and much dizziness ; the nervous system was greatly effected, the slightest causes producing high excitement and great disturbance.

Regarded the albuminuria as probably arising from functional derangement rather than organic disease, and had given a brief history of the case, as showing the value of tannin and the cathartics in such disease, since the albumen has nearly or quite disappeared, and the anasarca is very rapidly improving. The induction of premature labor would be regarded as one of the most important points to be decided. The reasons in this case were ample, and the result highly satisfactory, but since this is not the point under consideration would not occupy the time in relating them.

*Dr. Ring* stated that the case of Bright's disease reported by Prof. White, reminded him of two cases of *Uræmic* poisoning that had occurred in his practice during the present year, and which he regarded as of sufficient interest to be worth reporting to the Society. He then read the following :

On the 30th of January last, was sent for to visit Mrs. J. D. aged 52. She had been ailing for several days, which she attributed to exposure, and taking cold ; she was anasarcous ; the urine suppressed and albuminous ; bowels costive ; pulse 52, irregular and weak ; palpitation of the heart and oppression at the præcordiæ ; constant headache and at night delirious and restless ; ordered for her a cathartic of calomel and jalap ; a diuretic mixture of tinct. scillæ, spts. ether nit. wine colch. and cold water to the head. The next day found that the cathartic powder, although a small one, had operated well; she had spent a restless night, and the urine not much increased; I ordered the cold water continued to the head, it being pleasant to her ; the diuretic continued ; with sul. morphia $\frac{1}{8}$ gr. at bed-time, to procure rest and sleep ; the cold water was discontinued the next day ; with the morphia at night and the diuretic, the more urgent symptoms gradually disappeared in ten days, and the urine was clear of albumen after three weeks; she took nothing more except ferri sulphas and quinia as a tonic, until she was convalescent ; she is now in good health. At my first visit I was apprehensive of her having convulsions, and had many fears as to the result of the case.

The second case occurred in July last, Mr. F. S. aged 38. He had not felt well for a month previous to the 11th of July, when I visited, and found him with general dropsy, with an almost entire suppression of urine, which on being tested was found albuminous ; skin hot and dry ; pulse 100 per minute ; a constant and severe pain in the back part of the head, neck sore and stiff ; his respiration was short and oppressed, and it was difficult for him to lie down at all, so much effusion was there in the abdomen.

The treatment was the same as in the first case. The cathartic worked powerfully, and his bowels were somewhat purged for three days after ; by the use of the diuretic before mentioned, to which tinc. digitalis was added, the urine was increased, but still not free ; he took on the second and third day of my attendance, $\frac{1}{8}$ gr. sul. morphia at bedtime, but he thought it made him feel worse instead of better ; early on the morning of the 17th of July, Mr. S. had a convulsion ; I saw him soon after, and gave him a dov. powder, and sent for Prof. J. P. White in consultation ; before the Prof. arrived, Mr. S. had three fits, his pulse was about 112 per minute, and he was unconscious between them; the following prescription, was ordered:—Quinia sul. grs. xvi. morphia sul. grs. ii. spts. etheris nitrici ℥ i—M. Teaspoonful to be given every three hours.

At 10 A. M. the next day, we learned that he had five convulsions after we saw him, the last being at 2 o'clock in the night, the pulse was 85 per minute and less fever, but he was still quite unconscious ; medicine continued. July 19th, he slept well last night, this morning his consciousness has returned, and the pain has left his head; the urine is now free; he is much better. The medicine was continued for a week longer in gradually diminished doses. He made a good recovery, the only medicine given was iron and quinia, taken to restore his strength and appetite. He now has good health and is able to work at his trade. The result in this case was to us very gratifying. Among all the modes of practice and great number of remedies that have been used in these cases and puerperal convulsions, this practice may be worth remembering, and worthy of another trial. Of course one case is not expected to be worth much of itself, and farther experience may confirm or disprove the value of this method of treatment.

*Dr. Rochester* desired to make a slight comment upon the very interesting cases reported by Dr. Ring, especially with reference to the conclu sions drawn by Dr. Ring, upon the results of different modes of treatment. In the first place there was no proper analogy between these cases and those presented by Dr. White and himself. Dr. Ring's patients both had acute suppression of urine, a condition full of danger, and sure to be succeeded, unless speedily relieved, by fatal uræmia, but there was no evidence that they were cases of fatty or granular degeneration of the kidney. The presence of albumen in the urine was no proof of this. Respecting the novelty of the opium treatment, Dr. Rochester must remind Dr. Ring, that in puerperel eclampsia, opium in full doses had many advocates, especially before chloroform was known, and was used to prevent a return of the paroxysms. He mentioned this not to condemn Dr. Ring's treatment, which the event proved to have been highly judicious, but to show that Dr. Ring's treatment was by no means new or unprecedented.

*Dr. Ring* replied to Dr. Rochester, that he did not report his cases as Bright's disease, but uræmic poisoning. In regard to the treatment, it is unlike that usually recommended by authors.

*Prevailing Diseases.*—Dr. Strong spoke of the prevalence of whooping cough.

*Dr. Rochester* reported a fatal case of cholera occurring near the International Ferry, and spoke of other cases not so severe seen elsewhere which recovered.

*Dr. Wyckoff* had seen two or three cases of sporadic cholera, one near the residence of the late Dr. Treat; one at Sulphur Springs, supposed to be caused by eating pork. Calomel and opium controlled the disease.

*Dr. Samo* presented Treasurer's Semi-Annual Report. Voted that the President, Vice President and Secretary audit the accounts.

*Prof. Eastman*, in behalf of the Faculty of the University of Buffalo, presented the Association, the *United States Army Medical Statistics*, in two finely bound vols.

Voted that the thanks of this Association be tendered the Faculty for the valuable donation to its library. Adjourned.

J. F. MINER, *Sec'y.*

